# Inhibitory Activities of Momilactones A, B, E, and 7-Ketostigmasterol Isolated from Rice Husk on Paddy and Invasive Weeds

**DOI:** 10.3390/plants8060159

**Published:** 2019-06-07

**Authors:** Nguyen Van Quan, Tran Dang Xuan, Hoang-Dung Tran, Nguyen Thi Dieu Thuy

**Affiliations:** 1 Division of Development Technology, Graduate School for International Development and Cooperation (IDEC), Hiroshima University, Higashi Hiroshima 739-8529, Japan; nguyenquan26@gmail.com (N.V.Q.); dieuthuykttb@gmail.com (N.T.D.T.); 2 Faculty of Biotechnology, Nguyen Tat Thanh University, 298A-300A Nguyen Tat Thanh Street, Ward 13, District 4, Ho Chi Minh 72820, Vietnam

**Keywords:** Allelopathy, phytoalexins, momilactones, weed control, lettuce, barnyard grass, tall goldenrod

## Abstract

Rice husk has been exploited as a potential source of allelochemicals. In this study, four bioactive compounds including momilactone E (ME), 7-ketostigmasterol (7KS), momilactone A (MA), and momilactone B (MB) were isolated by column chromatography (CC) to yield 2.7, 0.3, 11.7, and 8.3 mg/kg rice husk, respectively. The structures of the isolated compounds were identified and confirmed by spectroscopic techniques consisting of ^1^H and ^13^C nuclear magnetic resonance (NMR), electrospray ionization mass (ESI), high-resolution mass spectrometry (HR-MS) and infrared spectroscopy (IS). An advanced quantitative method for MA and MB was achieved to increase the detectable yields of MA and MB in rice husk to 51.96 and 42.33 µg/mL, respectively. The inhibitory activities of MA, MB, ME, and 7KS were examined on lettuce (*Lactuca sativa*), barnyard grass (*Echinochloa crus-galli*), and tall goldenrod (*Solidago altissima*) in bioassays. The allelopathic activities of ME and 7KS were compared with those of potent phytoalexin momilactones A (MA) and B (MB), and the standard *p*-hydroxybenzoic acid (*p*HA). Results showed that both MA and MB exhibited stronger inhibitory activity than ME and 7KS. MB exerted greater inhibitions than MA but the mixture of MA and MB (1:1, *v*/*v*) possessed a similar level of inhibition to MB. On the other hand, although ME and 7KS presented non-significant inhibition, their mixture of ME-7KS (1:1, *v*/*v*) displayed a remarkable inhibition on the growth of *S. altissima*. Findings of this study revealed that MA, MB, and the mixture ME-7KS had the potential to control the invasive plant *S. altissima* and the noxious paddy weed *E. crus-galli* in vitro, but their mode of actions should be further investigated.

## 1. Introduction

Allelopathy is defined as a natural phenomenon when a plant produces and liberates phytochemicals into the environment [1]. Subsequently, the substances may inhibit or stimulate the growth of ambient species [2]. In agriculture, the application of allelopathy in weed management is not a novelty. The use of either plant products or derivatives of bioactive compounds from plants has increasingly become common, owing to their eco-friendly sustainable agricultural characteristics [3]. Mimosine, a potent allelochemical derived from *Leucaena leucocephala* and *Mimosa* spp., is a typical pattern of the utilization of a natural product which reduced weed emergence but increased rice yield [4]. As mimosine is effective for weed inhibition and is easily degradable, the synthesis of mimosine derivatives to develop novel pesticides is promising [4]. Among agricultural by-products which can reduce weed emergence, rice husk has been reported to possess promising allelochemicals [5]. In addition, the application of rice husk and rice bran were effective in paddy weed management [6]. The incorporation of rice husk in paddy field also increased approximately 20% of rice yield [5,7]. Moreover, the allelopathic capability of rice husk extract was extensively examined in a laboratory, greenhouse and paddy field [8]. There was a number of allelochemicals isolated from rice husk, including phenolics, fatty acids, and momilactones [3,9,10]. 

Momilactones A (MA) and B (MB) were first time-isolated and identified from rice husk by Kato et al. [9]. Among biological activities, MA and MB have been known as potent phytoalexins and allelochemicals [3,11,12], but recent researches show that they are also diabetes inhibitors [13,14]. Subsequently, momilactone C (MC) [15] and momilactone D (MD) [16] were identified in rice husk and root, respectively. However, only MC exhibited an effective inhibition on germination of *L. sativa* [15] whilst MD displayed a weak anti-inflammatory activity compared to MA by using nitric oxide production models [16]. Notably, in the research of Cho et al. [16], authors isolated a pimarane diterpenoid and named it momilactone E (ME). However, it was mistakenly defined because the constituent did not obtain any lactone moiety in its chemical structure. On the other hand, orizaterpenoid retrieved from rice hulls was reported to have a cytotoxic activity on P388 murine leukemia cells [6]. Structurally, orizaterpenoid consists of a lactone ring linked with the diterpenoid skeleton; therefore, such a compound can be considered as a momilactone. And, in this study, we suppose to name orizaterpenoid as momilactone E (ME). Meanwhile, 7-ketostigmasterol (7KS) was first time-isolated from rice husk [17]. The compound showed protective effects on human intestinal (Caco-2) cells [18], immune system [19], and antiviral (herpesvirus 1) activity [20]. Nevertheless, to date, no study on the allelopathic property of ME and 7KS was carried out.

The selection of test plants in the assessment of allelopathic activity of natural compounds is a crucial tool to evaluate the actual role and applicability of these compounds. Lettuce (*Lactuca sativa*) is widely used as a typical indicator plant, while barnyard grass (*Echinochloa crus-galli*), a major natural enemy of rice and crops in agricultural fields, is applied as an indicator weed in the allelopathic bioassays [5,21]. In addition, among invasive plants which currently cause trouble for environment, ecology, and crop production in Japan, tall goldenrod (*Solidago altissima*) is one of the most problematic. This plant species distributes throughout the country and affects many native species [22]. In order to preliminarily clarify the allelopathic role of MA, MB, ME, and 7KS, this research was conducted to examine the inhibitory effects of these compounds on the growth of noxious *E. crus-galli* and invasive *S. altissima*. The inhibition levels from different doses as well as the mixtures of the four compounds on the indicator plants were evaluated. 

## 2. Results

The inhibitory effects of the four isolated compounds on germinations of *L. sativa*, *E. crus-galli* and *S. altissima* are shown in Table 1. It was indicated that the suppressive influences of MA, MB, ME, and 7KS on germination of tested plants were differed. Of which, MB and the mixture of MA and MB (MAB) at a ratio 1:1 (*v*/*v*) exerted much stronger inhibition compared to ME and 7KS. Particularly, MAB showed significant inhibitions on the germination of *L. sativa* (IC_50_ = 327.20 µg/mL), *E. crus-galli* (IC_50_ = 28.26 µg/mL), and *S. altissima* (IC_50_ = 23.97 µg/mL). The IC_50_ values of MA (on *L. sativa*), ME, *p*-hydroxybenzoic acid (*p*HA), and the mixture of ME-7KS (on *L. sativa* and *E. crus-galli*), as well as 7KS (on all assays) were not calculated for *L. sativa* assay when the germination rate was 100% at all treated concentrations.

As shown in Table 1, ME1 showed a similar inhibition to the positive control *p*HA while other compounds and mixtures exhibited greater suppression on germination of *S. altissima*. The IC_50_ values of MA, MB, and MAB were 119.80, 20.36, and 23.97 µg/mL, respectively. Besides, the mixture ME-7KS (IC_50_ = 358.30 µg/mL) also expressed notable suppressions on the *S. altissima* germination. The mixture MAB presented a similar level of inhibition on *S. altissima* compared with MB. IC_50_ values of MB and MAB were approximately 5- and 22-fold greater than those of MA and ME-7KS, respectively, which indicated that MB may play a crucial role in the allelopathic activity.

The inhibitory effects of MA, MB and their mixture MAB on growths of *L. sativa* and *E. crus-galli* are shown in Table 2. Of which, MB and the mixture MAB showed similar levels of suppression and it was much greater than either MA or the control *p*HA. MA, MB and MAB inhibited root and shoot elongations of *E. crus-galli* more strongly than those of *L. sativa*. In contrast, the positive control *p*HA showed greater effects on *L. sativa* than *E. crus-galli* (Table 2). 

In Table 3, the inhibitory activity of MA, MB, ME, 7KS and their mixtures were compared at two concentrations 400 and 1000 µg/mL. In general, the inhibitory levels of MA, MB, as well as the mixture MAB were greater than ME, 7KS and the mixture ME-7KS. At 400 µg/mL, the germination and growth of *E. crus-galli* and *S. altissima* were almost suppressed, although *L. sativa* was inhibited at a lower level (Table 3). In contrast, even at 1000 µg/mL, shoot length of *L. sativa* was strongly promoted by ME, 7KS, and ME-7KS, whilst no effect on germination of both *L. sativa* and *E. crus-galli* was observed. Although the inhibitory effects of ME, 7KS and the mixture ME-7KS on germination of *S. altissima* were significantly stronger than on that of *L. sativa* and *E. crus-galli*, MA, MB and the mixture MAB recorded much greater inhibition on germination and growth of all tested plants (Table 3).

In this study, the quantitative methods for MA and MB by HPLC showed a significantly greater quantity of the two compounds in rice husk as compared with the previous study [13] (Table 4). Of which, the use of column type and flow rate were modified to enhance the detective level of both MA and MB to quantify much greater amounts of MA and MB (Table 4).

In this study, the amounts of MA and MB in rice husk were calculated as 51.96 µg/mL and 42.33 µg/mL, respectively, which were 3.2- and 4.6-fold higher than those of MA and MB quantified by the previous method [13]. The principal factor was attributed to the use of a Sep-Pak C18 cartridge in preparation of husk extract. By this application, some other components were firstly eliminated by 50% aqueous methanol, then the purer MA and MB were obtained from the 100% methanol solution. The other factor might be HPLC column length and flow rate. The use of a shorter analytical column (150 mm) and a slight increase in flow rate (0.5 mL/min) might influence the sensitivity of the HPLC system by enhancing LOD (0.05 ng/mL) and LOQ (0.14 ng/mL) of MA. The result provided an efficient option for selecting the most appropriate method of sample preparation and quantification of MA and MB.

## 3. Discussion

MA and MB have been admitted as plant growth-inhibitors and phytoalexins in rice plant organs and rice husk for more than 40 years, thus both MA and MB are believed to play a crucial role in protecting rice from multiple natural enemies including weeds and pathogens [6,23,24,25]. This study confirmed and specially emphasized the potent biological activity of MB in the growth restraint of some indicator species. To the best of our knowledge, this is the first time the growth inhibition of the mixture MAB has been investigated. MB exerted a stronger inhibition on plant growth than MA, and a mixture of MA and MB (1:1, *v*/*v*) did not show stronger effects than the solo treatment by MB (Table 2). The result can be construed by the exclusive structure of MB. A bridge -O- between C-3 and C-20 attached with a hydroxyl group in MB structure may bring about potent herbicidal activity compared with MA [13,25]. This finding may provide a new approach in the green production of herbicides which is environmentally friendly and cost saving. The use of mixture MA and MB did not show stronger inhibition than the individual MB did, thus the search for single constituents with strong bioactive effects to develop natural herbicides should be targeted. The application of a solo compound is apparently easier and lower cost than a mixture of many constituents. Additionally, the improved method for sample purification and HPLC analysis from this study increased the quantifying effectiveness of momilactones A and B by 3–4 times (Table 4), which helped determine more correctly the real contents of these active compounds in rice. 

For the other consideration, ME and 7KS were the first investigated for the allelopathic activity. While ME portrayed an exceptional stimulation on root growth of *L. sativa* and a minor enhancement on root and shoot elongations of *E. crus-galli*, it showed a considerable inhibition on germination of *S. altissima*. Given the synergistic effect of a mixture ME and 7KS (1:1, v/v) on the suppression of root and shoot lengths of *E. crus-galli* and germination of *S. altissima*, 7KS might possess a promising inhibitory effect on the growth of these indicator plants (Table 3). In fact, each plant has a specific way to respond with both abiotic and biotic stresses from the habitat. It depends on various factors including dose and intensity of factors [26] as well as the defense mechanism of a plant itself [27]. Presently, a large number of allelochemicals has been elucidated belonging to phenolics, flavonoids, terpenoids, alkaloids, and their derivatives [28]. The earlier study also manifested the potential role of sterols in the allelopathic activity of plants [29]. However, information of natural products being allelopathic on different plants has not been provided sufficiently. This study was the first to demonstrate the difference in allelopathic effects of a terpenoid (ME, orizaterpenoid) and a sterol (7KS, 7-ketostigmasterol) isolated from rice husk on the growth of various indicator plants including *L. sativa*, *E. crus-galli*, and *S. altissima*.

The most significant finding of this study was the inhibitory effect of active compounds from rice husk on the germination of tall goldenrod (*Solidago altissima*), a harmful invasive plant. Several studies were implemented to evaluate the germination of *S. altissima* seeds, however, most of them used sand or soil as the media [30,31]. For the current study, the germination of *S. altissima* was successfully assayed under the laboratory condition by using agar gel (0.5%) as a growth media. The method was designed in order to ensure the shortest time and the highest efficiency of plant germination. In nature, *S. altissima* grows very fast and wildly distributes in miscellaneous habitats [32] such as abandoned fields, edges of rice-fields, and pavements. It strongly competes with other plants in the habitat due to the tenacious rhizome system which can regenerate to form a new individual [33]. Additionally, *S. altissima* seed has a powerful dispersal ability thanks to its peculiar structure (wind spread) and a vast number of seeds. In Japan, *S. altissima* does not only affect the landscape, agricultural fields, and social infrastructures but also influences the health of humans and animals. There is an unofficial report that seeds of *S. altissima* when spreading in the air, may cause respiratory allergies in human and pets. The allelopathic results in this study indicated that the germination of *S. altissima* was the most susceptible by momilactones and was potentially inhibited by the mixture of ME-7KS. In addition, the active compounds besides MA, MB, ME, and 7KS isolated from rice husk and their derivatives should be further examined on harmful weeds and invasive species to improve the potential use of rice husk for weed management. The synthesis of derivative constituents derived from MA, MB, ME, and 7KS might have potential to develop novel and environmentally safer herbicides.

## 4. Materials and Methods

### 4.1. Materials

Rice husks of *Oryza sativa* (var. Koshihikari) were collected from rice mills in Higashi-Hiroshima, Japan in September 2016. Lettuce (*Lactuca sativa*) and barnyard grass (*Echinochloa crus-galli*) seeds were purchased from the Japan Agriculture, Hiroshima, Japan; and P2 and Associates Inc., Fukuoka, Japan, respectively. Meanwhile, tall goldenrod (*Solidago altissima*) seeds were collected in fields at 34.39°N, 132.74°E, Higashi-Hiroshima, Japan in November 2017. The dormancy of *E. crus-galli* and *S. altissima* was broken by keeping them in a freezer for 2 weeks at −20 °C to achieve the maximum germination rate. Pure standard compounds were obtained from previous studies of our laboratory [13,17].

### 4.2. Extraction and Isolation of Active Compounds from Rice Husk

**Figure 1:**
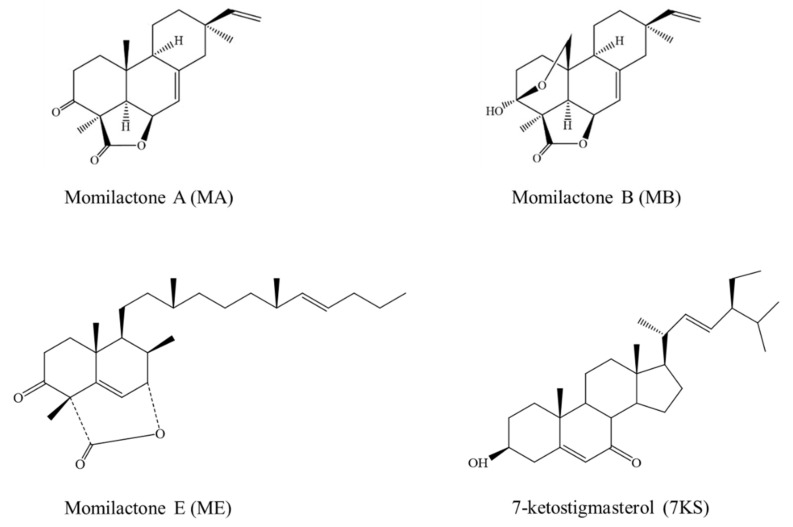
Chemical structures of MA, MB, ME, and 7KS.

The extraction and isolation of the four pure compounds MA, MB, MD, and 7KS from rice husk were described previously [17]. In brief, 30 kg of dried husk was extracted by methanol for 2 weeks at room temperature. The crude methanolic extract (485 g) was then separated by liquid–liquid extraction method using hexane, ethyl acetate (EtOAc) and water solvents. The resulting EtOAc extract (350 g) was subjected to repeated column chromatography over 60 Å pore size–silica gels (60–100 mesh in a 5 × 60 cm column followed by 200–400 mesh in a 2 × 50 cm column). Four pure compounds were yielded including momilactone A (MA, 350 mg), momilactone B (MB, 200 mg), orizaterpenoid (80 mg) that was defined as momilactone E (ME), and 7-ketostigmasterol (7KS, 10 mg), see Figure 1. The structures of the four isolated compounds were identified and confirmed by spectroscopic techniques consisting of ^1^H and ^13^C nuclear magnetic resonance (NMR, Bruker DRX-500 spectrophotometer, Bruker India Scientific Pvt. Ltd., New Delhi, India), electrospray ionization mass (ESI, LTQ Orbitrap XL mass spectrometer, Thermo Fisher Scientific, CA, USA), high-resolution mass spectrometry (HR-MS, 6545Q-TOF LC/MS, Agilent technology, Santa Clara, CA, USA), and infrared spectroscopy (IS, Shimadzu 8201 PC, Shimadzu Corporation, Kyoto, Japan). The spectroscopic methods followed the previous studies [12,13,17] and data were compared with those in the literature [17]. 

### 4.3. Allelopathic Bioassays

**Figure 2:**
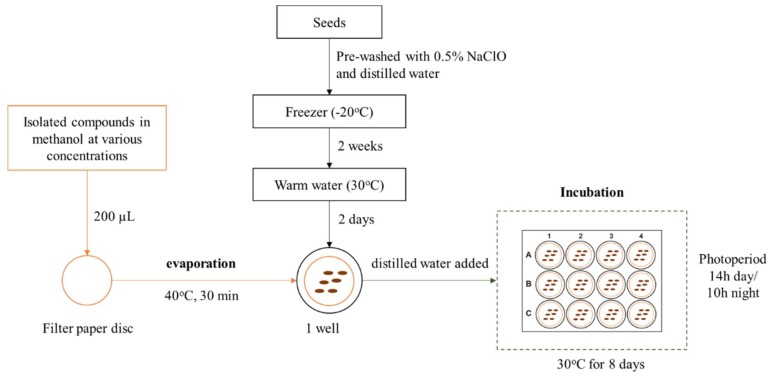
General experimental design of the in-vitro allelopathic assay.

Seeds of *L. sativa*, *E. crus-galli* and *S. altissima* were soaked in 0.5% aqueous sodium hypochlorite for 20 min and washed thoroughly with distilled water. *S. altissima* seeds were then dried by an oven at 35 °C for 4 days and then kept in a fridge at −20 °C for 2 weeks. Before experiments, selected healthy seeds were incubated in 300 mL of distilled water (30 °C) for 2 days. Germination and growth of the three species were conducted using Nunc™ 12-well plates (Thermo Fisher Scientific, Jiangsu, China) in a growth chamber (Biotron NC system, Nippon Medical & Chemical Instrument, Co. Ltd., Osaka, Japan) with a photoperiod of 14h day/10h night at 30 °C for 8 days (Figure 2). 

The four isolated compounds MA, MB, ME, and 7KS were dissolved in methanol at different dilutions. An aliquot of 0.2 mL of tested solution was penetrated to a filter paper disc (20 mm diameter) followed by evaporating in an oven at 40 °C for 30 min to subtract the effect of methanol on bioassay. Subsequently, a treated disc was placed into the corresponding well of the 12-well plate for the bioassay (Figure 2). Pure methanol was used as a negative control, while *p*-hydroxybenzoic acid (*p*HA) was a positive control. Each experiment was repeated three times. The germination rate of all species was determined, and root and shoot lengths of *L. sativa* and *E. crus-galli* were recorded. The inhibitory and stimulatory percentages were calculated over the negative control. The IC_50_ value represented a concentration that provided 50% inhibition and was calculated by a method of Fukuta et al. [24]. Based on the preliminary screening result and a limitation of isolated amount, the pure 7KS was used in *L. sativa* assay only.

For the allelopathic assay on *L. sativa*, a total of eight seeds were laid on the treated disc in each well. Afterwards, an amount of 0.4 mL of distilled water was added, and the plate was covered by wrapping paper prior to incubating in a growth chamber. In the case of *E. crus-galli* and *S. altissima*, each well of the Nunc™ 12-well plate was at first lined by a layer of 0.5% agar gel (1 mL), and then a treated paper disc was put on the surface followed by pipetting 0.2 mL of distilled water. A total of 8 seeds of *E. crus-galli* and 20 seeds of *S. altissima* were used in each trial. 

### 4.4. Quantification of Momilactones A and B by HPLC

In this study, the quantitative methods for MA and MB by HPLC were advanced as compared with a previous study described by Quan et al. (2019) [13]. Rice husk (100 g) was extracted with 200 mL of methanol for 1 week. The obtained methanolic extract was mixed with hexane in a separatory funnel for 2 h. Afterward, the methanolic phase was filtered and reconstituted by a vacuum evaporator (Rotavapor® R-300, Nihon Buchi K.K., Tokyo, Japan). The extract of rice husk was homogenized in 50% aqueous methanol and loaded into a Sep-Pak® Plus C18 cartridge (Waters Cooperation, Milford, MA, USA). The cartridge was washed with 2 mL of 50% methanol and then eluted with 10 mL of 100% methanol. Subsequently, the methanol solutions were combined and adjusted into the concentration of 10 mg/mL which was used for HPLC analysis. Five microliters of the extract were injected into the HPLC system comprising PU-4180 RHPLC pump, LC-Net II/ADC controller, and UV-4075 UV/Vis detector (Jasco, Tokyo, Japan). The stationary phase was Waters Spherisorb ODS2 column (10 µm, 150 mm × 4.6 mm i.d.) acquired from Waters Cooperation, Milford, MA, USA. The mobile phase consisted of 0.1% trifluoroacetic acid in 70% aqueous acetonitrile. The operation time was set for 25 min with a flow rate of 0.5 mL/min. The wavelength of 210 nm was used for detecting appearances of MA and MB. The quantification of MA and MB in rice husk extract was calculated according to the retention times and peak areas of the standards MA and MB with the sample.

The sensitivity of the HPLC system was expressed as limits of detection (LOD) and limits of quantitation (LOQ) by linear regression analyses of peak areas against concentrations of isolated momilactones A and B.

### 4.5. Statistical Analysis

All bioassays were carried out thrice in a completely randomized design (*n* = 3). Data were analyzed by the Minitab 16.0 software (Minitab Inc., State College, PA, USA). Results were displayed as means ± standard errors (SE). The IC_50_ value was the amount required to inhibit 50% germination or growth of the test plants. Significant differences among assays were evaluated by one-way ANOVA using Tukey’s test at *p* < 0.05.

## 5. Conclusions

Findings of this study showed that MA, MB, ME, and 7KS were plant growth-inhibitors in rice husk, although the levels of inhibition of these compounds varied. Among the four compounds, MB was the most inhibitory, followed by MA. Both ME and 7KS exhibited lower suppressive effects than MA and MB. MA, MB, and the mixture MAB had potential to inhibit the growth of both barnyard grass (*E. crus-galli*) and tall goldenrod (*S. altissima*), whilst only the mixture of ME-7KS showed strong inhibition towards the emergence of *S. altissima*. This study also achieved an advanced quantitative method to detect MA and MB in rice husk by 51.96 and 42.33 µg/mL, respectively, or 3.16- and 4.58-fold higher as compared with our previous research. MA, MB, and the mixture ME-7KS showed promise in controlling the harmful paddy weed *E. crus-galli* and the invasive *S. altissima* in vitro, but their modes of action should be investigated further.

## Figures and Tables

**Table 1:** IC_50_ value (µg/mL) for inhibitory effect of isolated compounds on germination rate of tested plants.

Sample	*L. sativa*	*E. crus-galli*	*S. altissima*
MA	nd	229.67 ± 1.20 b	119.80 ± 11.50 b
MB	178.46 ± 0.03 b	31.88 ± 0.18 a	20.36 ± 0.91 a
MAB	327.20 ± 27.50 a	28.26 ± 3.05 a	23.97 ± 1.89 a
ME	nd	nd	1162.50 ± 14.10 d
7KS	nd	nd	nd
ME-7KS	nd	nd	358.30 ± 8.39 c
*p*HA	nd	nd	1074.20 ± 66.60 d

Means ± standard error. Means within a column followed by similar letters are not significantly different by Turkey’s test (*p* < 0.05); nd = not determined; MA, momilactone A; MB, momilactone B; MAB, mixture of momilactones A and B (1:1, *v*/*v*); ME, momilactone E; 7KS, 7-keto-stigmasterol; ME-7KS, mixture of momilactone E and 7-keto-stigmasterol (1:1 *v*/*v*); *p*HA, *p*-hydroxybenzoic acid.

**Table 2:** IC_50_ value (µg/mL) for inhibitory effect of MA and B on growth of *L. sativa* and *E. crus-galli*.

Sample	*L. sativa*		*E. crus-galli*	
	RL	SL	RL	SL
MA	348.00 ± 11.30 b	388.20 ± 10.70 b	147.60 ± 0.37 b	123.87 ± 1.78 b
MB	6.49 ± 0.04 a	58.64 ± 0.99 a	4.00 ± 0.03 a	4.46 ± 0.05 a
MAB	5.63 ± 0.05 a	63.77 ± 0.64 a	5.13 ± 0.05 a	4.47 ± 0.05 a
*p*HA	820.57 ± 9.8 c	1453.30 ± 48.10 c	936.66 ± 4.49 c	1464.6 ± 40.30 c

Data presented a means ± standard error. Means within a column followed by similar letters are not significantly different by Turkey’s test (*p* < 0.05); RL, root length; SL, shoot length; MA, momilactone A; MB, momilactone B; MAB, mixture of momilactones A and B (1:1, *v*/*v*); *p*HA, *p*-hydroxybenzoic acid.

**Table 3:** Comparative allelopathic effects (%) of isolated compounds on growth of tested plants.

Sample	Concentration (µg/mL)	*L. sativa*			*E. crus-galli*				*S. altissima*	
		GR	RL	SL		GR	RL	SL		GR
Methanol		(8/8) ^a^ 0.0	(1.95) ^b^ 0.0	(0.76) ^c^ 0.0		(8/8) 0.0	(4.53) 0.0	(3.03) 0.0		(13/20) 0.0
MA	400	0.0	52.38	50.67		78.57	95.96	96.70		86.75
MB		100.00	94.71	84.59		100.00	100.00	100.00		100.00
MAB		58.33	91.82	82.82		100.00	100.00	100.00		100.00
ME	1000	0.0	+92.68	6.61		0.0	+16.48	+8.01		39.53
7KS		0.0	+60.93	13.65		nd	nd	nd		nd
ME-7KS		0.0	+93.00	5.71		0.0	13.33	29.52		94.66
*p*HA		0.0	63.14	37.01		0.0	58.46	36.12		44.66

Data shows inhibition percentages over the control; +, stimulation percentages over the control; MA, momilactone A; MB, momilactone B; MAB, mixture of momilactones A and B (1:1, *v*/*v*); ME, momilactone E; 7KS, 7-keto-stigmasterol; ME-7KS, mixture of momilactone E and 7-keto-stigmasterol (1:1, *v*/*v*); *p*HA, *p*-hydroxybenzoic acid; GR, growth rate; RL, root length; SL, shoot length; nd, not determined. Numbers in parentheses are baselines of negative control; ^a^, number of germinated seeds; ^b,c^, root and shoot lengths (cm).

**Table 4:** Advanced development of the quantitative methods by HPLC for MA and MB.

Parameters	This Study	Previous Study [13]
Sep-Pak C18 cartridge	Used	Not use
HPLC column	10 µm, 150 mm × 4.6 mm i.d.	10 µm, 250 mm × 4.6 mm i.d.
Flow rate	0.5 mL/min	0.4 mL/min
LOD	0.05 ng/mL (MA), 0.48 ng/mL (MB)	0.43 ng/mL (MA), 0.18 ng/mL (MB)
LOQ	0.14 ng/mL (MA), 1.46 ng/mL (MB)	1.31 ng/mL (MA), 0.54 ng/mL (MB)
Quantity of MA	51.96 µg/mL	16.44 µg/mL
Quantity of MB	42.33 µg/mL	9.24 µg/mL

HPLC, high performance liquid chromatography; LOD, limit of detection; LOQ, limit of quantification; MA, momilactone A; MB, momilactone B.

## References

[1] Rice E.L., Cooper-Driver G.A., Swain T., Conn E.E. (1985). Allelopathy—An Overview. Chemically Mediated Interactions between Plants and Other Organisms. Recent Advances in Phytochemistry.

[2] Xuan T.D., Elzaawely A.A., Deba F., Fukuta M., Tawata S. (2006). Mimosine in Leucaena as a potent bio-herbicide. Agron. Sustain. Dev..

[3] Khanh T.D., Xuan T.D., Chung I.M. (2007). Rice allelopathy and the possibility for weed management. Ann. Appl. Biol..

[4] Xuan T.D., Tawata S., Khanh T.D., Price A., Kelton J. (2013). Herbicidal activity of mimosine and its derivatives. Herbicides—Advances in Research.

[5] Xuan T.D., Tsuzuki E., Tawata S., Khanh T.D. (2004). Method to determine allelopathic potential of crop plants for weed control. Allelopathy J..

[6] Chung I.M., Ali M., Hahn S.J., Siddiqui N.A., Lim Y.H., Ahmad A. (2005). Chemical constituents from the hulls of *Oryza sativa* with cytotoxic activity. Chem. Nat. Compd..

[7] Esa N.M., Ling T.B., Peng L.S. (2013). By-products of rice processing: An overview of health benefits and applications. J. Rice Res..

[8] Khang D.T., Anh L.H., Ha P.T.T., Tuyen P.T., Quan N.V., Minh L.T., Quan N.T., Minh T.N., Xuan T.D., Khanh T.D., Trung K.H. (2016). Allelopathic activity of dehulled rice and its allelochemicals on weed germination. Int. Lett. Nat. Sci..

[9] Kato T., Kabuto C., Sasaki N., Tsunagawa M., Aizawa H., Fujita K., Kato Y., Kitahara Y., Takahashi N. (1973). Momilactones, growth inhibitors from rice, *Oryza sativa* L.. Tetrahedron Lett..

[10] Berendji S., Asghari J.B., Matin A.A. (2008). Allelopathic potential of rice (*Oryza sativa*) varieties on seedling growth of barnyardgrass (*Echinochloa crus-galli*). J. Plant Interact..

[11] Minh T.N., Xuan T.D., Ahmad A., Elzaawely A.A., Teschke R., Van T.M. (2018). Efficacy from different extractions for chemical profile and biological activities of rice husk. Sustainability.

[12] Minh T.N., Xuan T.D., Ahmad A., Elzaawely A.A., Teschke R., Van T.M. (2018). Momilactones A and B: Optimization of yields from isolation and purification. Separations.

[13] Quan N.V., Hoang-Dung T., Xuan T.D., Ahmad A., Dat T.D., Khanh T.D., Teschke R. (2019). Momilactones A and B are α-amylase and α-glucosidase inhibitors. Molecules.

[14] Quan N.V., Xuan T.D., Hoang-Dung T., Ahmad A., Khanh T.D., Dat T.D. (2019). Contribution of momilactones A and B to diabetes inhibitory potential of rice bran: Evidence from in vitro assays. Saudi Pharm. J..

[15] Tsunakawa M., Ohba A., Sasaki N., Kabuto C. (1976). Momilactone C, a minor constituent of growth inhibitors in rice husk. Chem. Lett..

[16] Cho J., Cha B., Min Lee S., Shrestha S., Jeong R., Sung Lee D., Kim Y., Lee D., Kang H., Kim J., Baek N. (2015). Diterpenes from the roots of *Oryza sativa* L. and their inhibition activity on NO production in LPS-stimulated RAW264.7 macrophages. Chem. Biodivers..

[17] Ahmad A., Xuan T.D., Minh T.N., Siddiqui N.A., Quan N.V. (2019). Comparative extraction and simple isolation improvement techniques of active constituents’ momilactone A and B from rice husks of Oryza sativa by HPLC analysis and column chromatography. Saudi Pharm. J..

[18] Alemany L., Laparra J.M., Barberá R., Alegría A. (2012). Evaluation of the cytotoxic effect of 7keto-stigmasterol and 7keto-cholesterol in human intestinal (Caco-2) cells. Food Chem. Toxicol..

[19] Laparra J.M., Alfonso-García A., Alegría A., Barberá R., Cilla A. (2015). 7keto-stigmasterol and 7keto-cholesterol induce differential proteome changes to intestinal epitelial (Caco-2) cells. Food Chem. Toxicol..

[20] Marinho R.d.S.S., Ramos C.J.B., Leite J.P.G., Teixeira V.L., Paixão I.C.N.d.P., Belo C.A.D., Pereira A.B., Pinto A.M.V. (2017). Antiviral activity of 7-keto-stigmasterol obtained from green Antarctic algae *Prasiola crispa* against equine herpesvirus 1. J. Appl. Phycol..

[21] Khanh T.D., Cong L.C., Chung I.M., Xuan T.D., Tawata S. (2009). Variation of weed-suppressing potential of Vietnamese rice cultivars against barnyardgrass (*Echinochloa crus-galli*) in laboratory, greenhouse and field screenings. J. Plant Interact..

[22] Fujii Y., Gopalakrishnakone P., Carlini C., Ligabue-Braun R. (2017). Toxic chemicals from invasive alien plants. Plant Toxins. Toxinology.

[23] Kato T., Tsunakawa M., Sasaki N., Aizawa H., Fujita K., Kitahara Y., Takahashi N. (1977). Growth and germination inhibitors in rice husks. Phytochemistry.

[24] Cartwright D.W., Langcake P., Pryce R.J., Leworthy D.P., Ride J.P. (1981). Isolation and characterization of two phytoalexins from rice as momilactones A and B. Phytochemistry.

[25] Fukuta M., Xuan T.D., Deba F., Tawata S., Khanh T.D., Chung I.M. (2007). Comparative efficacies in vitro of antibacterial, fungicidal, antioxidant, and herbicidal activities of momilactones A and B. J. Plant Interact..

[26] Xuan T.D., Shinkichi T., Khanh T.D., Chung I.M. (2005). Biological control of weeds and plant pathogens in paddy rice by exploiting plant allelopathy: An overview. Crop Prot..

[27] War A.R., Paulraj M.G., Ahmad T., Buhroo A.A., Hussain B., Ignacimuthu S., Sharma H.C. (2012). Mechanisms of plant defense against insect herbivores. Plant Signal Behav..

[28] Khanh T.D., Xuan T.D., Chung I.M., Tawata S. (2008). Allelochemicals of barnyardgrass-infested soil and their activities on crops and weeds. Weed Biol. Manag..

[29] Fischer N.H., Quijano L., Thompson A.C. (1985). Allelopathic agents from common weeds. The Chemistry of Allelopathy.

[30] Walck J.L., Baskin J.M., Baskin C.C. (1997). A comparative study of the seed germination biology of a narrow endemic and two geographically-widespread species of *Solidago* (Asteraceae). 1. Germination phenology and effect of cold stratification on germination. Seed Sci. Res..

[31] Meyer A.H., Schmid B. (1999). Seed dynamics and seedling establishment invading perennial *Solidago altissima* under different experimental treatments. J. Ecol..

[32] Sakata Y., Kaneko S., Hayano A., Inoue-Murayama M., Ohgushi T., Isagi Y. (2013). Isolation and characterization of microsatellite loci in the invasive herb *Solidago altissima* (Asteraceae). Appl. Plant Sci..

[33] Heath J.J., Kessler A., Woebbe E., Cipollini D., Stireman J.O. (2014). Exploring plant defense theory in tall goldenrod, *Solidago altissima*. New Phytol..

